# Surveillance optimisation to detect poliovirus in the pre-eradication era: a modelling study of England and Wales

**DOI:** 10.1017/S0950268820001004

**Published:** 2020-05-13

**Authors:** K. M. O'Reilly, N. C. Grassly, D. J. Allen, M. Bannister-Tyrrell, A. Cameron, A. I. Carrion Martin, M. Ramsay, R. Pebody, M. Zambon

**Affiliations:** 1 Department of Infectious Disease Epidemiology, London School of Hygiene & Tropical Medicine, London, UK; 2 Centre for Mathematical Modelling of Infectious Diseases, London School of Hygiene & Tropical Medicine, London, UK; 3 Department of Infectious Disease Epidemiology, MRC Centre for Outbreak Analysis and Modelling, St Mary's Campus, Imperial College London, London, UK; 4 Department of Infection Biology, London School of Hygiene and Tropical Medicine, London, UK; 5 Vaccine Centre, London School of Hygiene & Tropical Medicine, London, UK; 6 National Infection Service, Public Health England, London, UK; 7 Ausvet Europe, 3 Rue Camille Jordan, 69001 Lyon, France; 8 Ausvet, 5 Shuffrey Street, Fremantle, WA 6160, Australia; 9 European Program for Intervention Epidemiology Training (EPIET), European Centre for Disease Prevention and Control, (ECDC), Stockholm, Sweden

**Keywords:** Polio, surveillance, surveillance system, virology (human) and epidemiology

## Abstract

Surveillance for acute flaccid paralysis (AFP) cases are essential for polio eradication. However, as most poliovirus infections are asymptomatic and some regions of the world are inaccessible, additional surveillance tools require development. Within England and Wales, we demonstrate how inclusion of environmental sampling (ENV) improves the sensitivity of detecting both wild and vaccine-derived polioviruses (VDPVs) when compared to current surveillance. Statistical modelling was used to estimate the spatial risk of wild and VDPV importation and circulation in England and Wales. We estimate the sensitivity of each surveillance mode to detect poliovirus and the probability of being free from poliovirus, defined as being below a pre-specified prevalence of infection. Poliovirus risk was higher within local authorities in Manchester, Birmingham, Bradford and London. The sensitivity of detecting wild poliovirus within a given month using AFP and enterovirus surveillance was estimated to be 0.096 (95% CI 0.055–0.134). Inclusion of ENV in the three highest risk local authorities and a site in London increased surveillance sensitivity to 0.192 (95% CI 0.191–0.193). The sensitivity of ENV strategies can be compared using the framework by varying sites and the frequency of sampling. The probability of being free from poliovirus slowly increased from the date of the last case in 1993. ENV within areas thought to have the highest risk improves detection of poliovirus, and has the potential to improve confidence in the polio-free status of England and Wales and detect VDPVs.

## Introduction

Indigenous wild poliovirus has been not been reported within England and Wales since the 1970s [1]. The elimination of poliomyelitis was achieved largely through vaccination of children and adults, using both the oral and inactivated polio vaccines (OPV and IPV, respectively). The IPV was introduced in 1956 and was replaced by the OPV in 1962 where it was part of the routine immunisation programme. In 2004, the OPV was replaced by the IPV owing to the lower risk of vaccine-associated paralytic poliomyelitis (VAPP) cases. After the introduction of vaccination wild poliomyelitis cases quickly reduced in number; sporadic imported cases of wild poliomyelitis cases were reported within England and Wales until the 1980s, which emphases the need for high immunisation rates until polio is eradicated globally [2].

Across the decades from endemicity to elimination within England and Wales, surveillance for poliomyelitis has required adaptation. Global surveillance for poliomyelitis was developed in the 1990s within the Pan American Health Organization to detect cases, focussed within polio endemic settings. All cases of acute flaccid paralysis (AFP, the typical clinical presentation of poliomyelitis) in children <15 years should be investigated, and country surveillance rates have been used to determine an adequate surveillance system. AFP surveillance was instituted throughout the United Kingdom by 1991 where children <15 years presenting with AFP of any aetiology were investigated for poliomyelitis [1]. The reported AFP rate was ~0.38 per 100 000 and ~54% of cases had at least one stool sample collected for virology. Approximately 58% of AFP cases were discarded as polio and diagnosed as Guillain−Barre syndrome. The comparatively low AFP reporting rate has been consistent in subsequent years and reflects reporting rates within other high-income countries in Western Europe [3]. To further support the evidence base for the polio-free status of England and Wales, enterovirus surveillance (ENT) was included as part of the poliovirus submissions in the early 2000s, where children presenting with meningitis (a rarer clinical presentation of poliovirus) were tested for the presence of enterovirus infection, including poliovirus [1]. However, AFP and ENT surveillance will only detect clinical disease and as poliovirus infection is largely asymptomatic more appropriate tools are required. Internationally, environmental sampling (ENV) for poliovirus has been very useful in providing both evidence of elimination but also in detection of small outbreaks and otherwise undetected transmission in IPV vaccinated populations (where IPV protects against poliomyelitis but provides little mucosal immunity against infection) [4].

As eradication draws closer, surveillance for residual transmission and early indications of new importations and emergence events becomes increasingly important [5]. Each WHO regional office is carrying out a certification process for poliomyelitis eradication, where epidemiological evidence is reviewed to ascertain the polio-free status of each country [3]. One challenge is to assess and compare the available evidence of being polio-free given the different epidemiological surveillance activities and importation risk within each country. Additionally, vaccine-derived polioviruses (VDPVs) have increased in incidence since the removal of serotype 2 from OPV in 2016. VDPVs originate from the OPV vaccine but have acquired specific mutations that increase the probability of poliovirus infection resulting in paralysis, and easily spreads in unvaccinated populations. Although no VDPVs have been detected in England and Wales, it remains essential to have sufficient surveillance to detect any importation and transmission events.

We make the distinction between detection of wild poliovirus and VDPVs because wild infections now consist of only serotype 1, and a majority of VDPV infections are of serotype 2 with a lower probability of clinical disease. We assume that current surveillance activities continue and explore how introducing ENV surveillance can supplement current activities. Using a statistical framework, we aim to answer

1. Where in England and Wales should ENV surveillance be implemented to optimise detection of wild type and VDPVs?
2. How does ENV surveillance improve the evidence that England and Wales are free from wild type and VDPVs?

## Methods

Poliomyelitis cases are classified according to virus origin; wild-type poliomyelitis cases are those that have a close genetic relation to other wild-type viruses whilst vaccine-associated poliomyelitis cases have originated from the attenuated strain used in the OPV. In this analysis, we consider wild-type poliovirus, which now consist only of serotype 1, and VDPVs which we assume to be of serotype 2 [6]. We do not consider VAPP cases or transmission from immune-deficient VPDV shedders [7]. These considerations are in line with the England and Wales National Guidelines for Polio [8].

### Estimating the spatial variation in the potential for poliovirus circulation in England and Wales

Poliovirus circulation is defined as the sustained circulation through several chains of transmission of either wild type or VDPVs within a localised area of England and Wales as a result of importation of the index infection (or case). The potential for poliovirus circulation was assumed to be the combined effect of the importation rate and the probability of local virus circulation, and was estimated for each local authority (LA). We assume that poliovirus importation varies spatially within England and Wales according to localised international migration. Importation will be driven by international travel; either residents acquiring poliovirus while abroad or the arrival of international visitors. While the numbers of residents travelling internationally and the number of visitors is well documented at a country level, the sub-national location of both of these groups is not adequately reported. We make a simplifying assumption that the location of foreign-born nationals approximates the location of residents visiting countries and for visitors from each country. We focus on residents from countries that have reported either wild-type or VDPVs between 2015 and 2017 (Table 1).

**Table 1.** Countries that have reported either wild of VDPVs between 2015 and 2017 and the reported number of movements between England and Wales

Country	Population size (million)	Cases of wild poliovirus (2015–2017)	Incidence of wild poliovirus (2015–2017) (per million per year)	Cases of VDPVs (2015–2017)	Incidence of VDPVs (2015–2017) (per million per year)	Combined incidence (per million per year)	Travel to England and Wales (2016)	Visitors from England and Wales (2016)
Afghanistan	34.6	47	0.45	0	0	**0**.**45**	ND	15 351
Pakistan	193.2	82	0.14	3	0.01	**0**.**15**	65 776	552 833
Nigeria	186	4	0.01	2	0	0.01	100 904	183 807
Madagascar	24.9	0	0	10	0.13	0.13	ND	8289
Laos	6.8	0	0	11	0.54	0.54	ND	3032
DRC	78.7	0	0	22	0.09	0.09	ND	ND
Syria	18.4	0	0	74	1.34	1.34	ND	ND
Guinea	12.1	0	0	7	0.19	0.19	ND	ND
Myanmar	52.9	0	0	2	0.01	0.01	ND	15 287

ND, no data, presumed to be very low.

Data on the locality of foreign-born nationals are available from census data [9]. These data are reported at a LA level, consisting of 326 geographical units within England and 22 within Wales (348 in total). The travel patterns of UK residents and visitors to the UK are available from the International Passenger Survey (IPS) [10]. Only data for residents and visitors to Pakistan and India were available with sufficient accuracy, the remaining countries were grouped with other countries within a geographical region (West Africa, South East Asia, Middle East) and the numbers were adjusted according to the proportion of the region that reside in each country.

The probability of local poliovirus circulation (herein referred to as poliovirus circulation) was estimated. We assumed that for each LA the probability of circulation follows a binomial model with exposure being the number of movements between England and Wales and each country and the probability of circulation given introduction (supplement). Since 2004, the IPV vaccine was included in the routine immunisation schedule, as part of the ‘pentavalent’ vaccine, and the OPV was phased out. Circulation given introduction is a function of the assumed basic reproduction number and local pentavalent coverage. For LAs estimated to have a higher poliovirus risk, the relevant water company and, where possible, the likely sewage treatment works that would need to be sampled are provided.

### Estimating the probability of being free from poliovirus using surveillance data

Being ‘free’ from poliovirus is a distinct concept from elimination or eradication. Elimination is defined as the reduction to zero of the incidence of a specified disease in a defined geographical area and eradication is the permanent global reduction to zero of the incidence of infection. Being free from poliovirus refers to the incidence of infection being below a pre-specified threshold, and the threshold is informed by globally accepted indicators of surveillance. Whilst elimination was confirmed in England and Wales in the 1970s, surveillance is required to detect the re-emergence of polio should it be re-introduced. Comparison of different modes and efforts of surveillance can be subjective, and so to quantify the quality of evidence from surveillance, we estimate the probability of freedom from poliovirus [10].

**Fig. 1.**
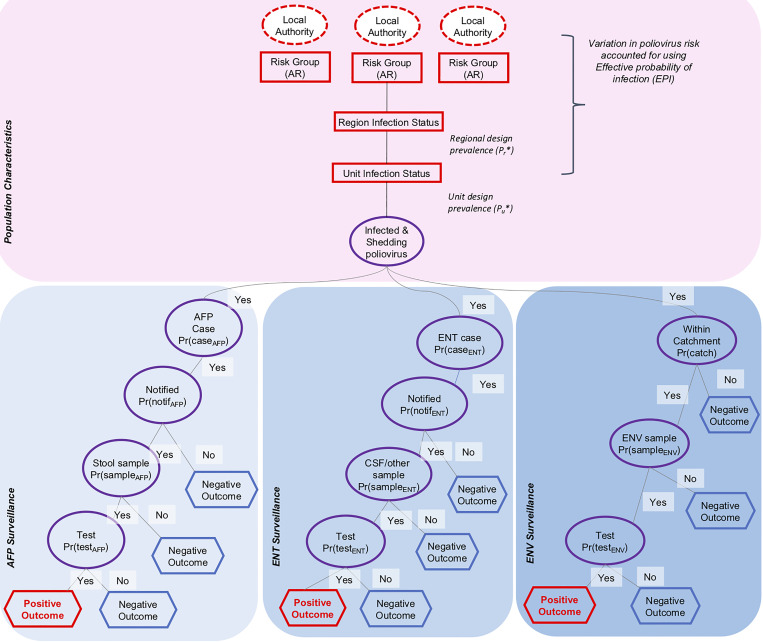
Scenario tree structure for acute flaccid paralysis (AFP), enterovirus (ENT) and environmental (ENV) surveillance in England and Wales. Dashed circles indicate category nodes, squares indicate infection nodes, circles indicate detection nodes and hexagons indicate outcome nodes.

We follow methods that have largely been developed in animal health [11, 12]. The population is divided into LAs and the surveillance system is divided into its constituent modes of surveillance (Fig. 1). We then determine a ‘design prevalence’, which is the prevalence of infection that the surveillance system is designed to detect. We use the standard surveillance indicator within polio eradication of one AFP case (all causes) per 100 000 individuals aged <15 years per year. As infection is likely to cluster (especially if an epidemic occurs), we include this by specifying the regional design prevalence of detecting at least one LA with poliovirus at the specified design prevalence. The combined effect of risk and design prevalence is included in the ‘effective probability of infection’ [12]. Each mode of surveillance (AFP, ENT and ENV) is then characterised by considering the sensitivity of detection at each stage of sampling. Each is briefly described in turn below, and parameter values are summarised in Table 2, and described in full in the supplement.

**Table 2.** Estimates of surveillance probabilities used in the scenario tree analysis

Surveillance	AFP		ENT		ENV	
Model inputs	Probability	Estimate (95% CI)	Probability	Estimate (95% CI)	Probability	Estimate (95% CI)
Infection – wild	Pr(case_AFP_)	0.00531 (0.00412–0.00668)	Pr(case_ENT_)	5.29×10^−5^ (4.07×10^−5^–6.74×10^−5^)	Pr(shedding)	0.80
Infection – VDPV		0.000567 (0.000281–0.000933)		As above		0.80
Notification	Pr(notif_AFP_)	0.9 (0.6–0.99)	Pr(notif_ENT_)	0.9 (0.6–0.99)	Pr(catchment)	varied
Sampling	Pr(sample_AFP_)	0.8 (0.5–0.95)	Pr(sample_ENT_)	0.5 (0.1–0.9)	Pr(sample)	0.80 (0.5–0.90)^a^
Test	Pr(test_AFP_)	0.97 (0.95–1.00)	Pr(test_ENT_)	0.97 (0.95–1.00)	Pr(test_ENV_)	0.97 (0.95–1.00)

The rationale behind the selected values are described in more detail in the Supplementary Material.

a Monthly sampling.

For AFP surveillance, the sensitivity is a product of the probability that an individual infected with poliovirus will develop symptoms consistent with AFP, which varies by serotype, the probability that an AFP case is admitted to a hospital and notified, and the probability that the case is correctly identified as poliomyelitis through collection and isolation of poliovirus in stool. This is summarised using the surveillance sensitivity per month (*CSe*_AFP_).

ENT surveillance arises from investigation of individuals accessing healthcare and from whom an enterovirus-positive specimen has been obtained. All NHS laboratories are requested to submit enterovirus-positive specimens for surveillance of poliovirus. ENT surveillance captures different clinical presentations, many of which are consistent with viral meningitis [13]. For infection with poliovirus, meningitis can occur in approximately 1% of clinical cases [14], with no available data on variation by serotype. In the model, we assume the notification rate for ENT is as high as for presentation with AFP. A majority of clinical samples collected by ENT surveillance consist of either stool, which has good sensitivity to detect poliovirus [13], or cerebral spinal fluid samples where the sensitivity of detecting enteroviruses is lower. In the UK between 2000 and 2011 enterovirus infections were detected in clinical samples, which included 5 032 cerebrospinal fluid samples and 2 394 gastrointestinal samples (that are most likely stool samples) [15], and 43% of all enterovirus infections were detected via gastrointestinal samples. Clinical specimens are usually tested for enterovirus RNA using polymerase chain reaction testing and has been demonstrated to be a useful method for detection in these sample types [16]. Where enterovirus RNA is detected, further laboratory investigations will aim to rule out poliovirus as the causative agent. In the model, we account for the variation in sensitivity of cerebrospinal and stool samples that are part of ENT surveillance by assuming that the sensitivity of individual clinical samples has a lower confidence bound in comparison to AFP surveillance (where all clinical samples are stool). In the model, the sensitivity of ENT surveillance over a period of one month is *CSe*_ENT_.

ENV surveillance is included in the framework by specifying whether each LA includes ENV. For those LAs with no ENV, the sensitivity of ENV to detect poliovirus is zero. As ENV surveillance is under development in England and Wales, we vary the frequency and location of ENV to explore the effect on surveillance sensitivity. The sensitivity of a sample is assumed to depend on the proportion of residents included in the sewage catchment, the probability that a sample contains poliovirus if an individual is shedding and the laboratory sensitivity which is thought to be high [17]. The sensitivity of AFP surveillance over a period of one month is *CSe*_ENV_.

The combined surveillance sensitivity of the system is calculated; *CSe* = 1 − (1 − *CSe*_AFP_)(1 − *CSe*_ENT_)(1 − *CSe*_ENV_). Using the principal of the negative predictive value of a test, the probability of being infection free within a given month can be calculated. The probability of being polio free was then estimated for each month from January 1993 to present day, with the addition of ENT surveillance in 1997, and assuming ENV in 2019. All the analyses were carried out in the software R (version 3.6.1.) and the code to replicate the analysis is available (https://github.com/kath-o-reilly/polio-FFI-UK).

## Results

### Spatial estimates of poliovirus risk in England and Wales

Between 2015 and 2017, Afghanistan, Pakistan and Nigeria reported cases of wild-type poliomyelitis while Pakistan, Nigeria, Madagascar, Laos, DR Congo, Syria, Guinea and Myanmar reported cases of VDPVs. Within Pakistan and Nigeria, more visits were made by residents of England and Wales to the country than visitors from each country (Table 1). For Afghanistan, Pakistan and Nigeria, a majority of visitors were visiting friends and relatives, supporting the assumption that their location will correlate with the location of foreign-born nationals.

**Fig. 2.**
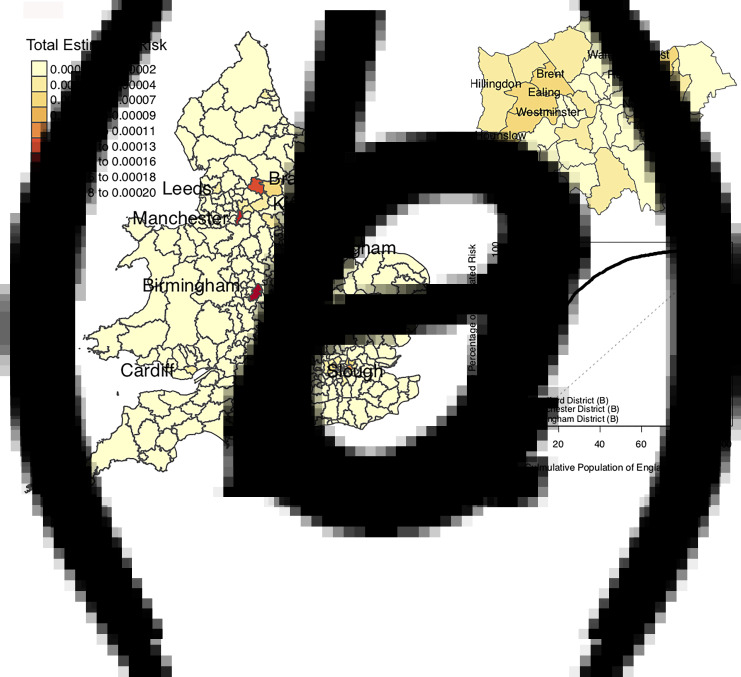
Estimated risk of poliovirus circulation in local authorities within (A) England and Wales, and (B) London. (C) The estimated risk within each local authority is ordered by reducing risk and compared to the cumulative percentage of the population to illustrate that 50% of estimated risk is focussed within <20% of the population.

Within England and Wales, the locality of long- and short-term residents born outside of England and Wales varies spatially and are often focussed within cities and associated conurbations, especially Birmingham, Bradford, London and Manchester. Coverage of the pentavalent vaccine varies across England and Wales, with an average of 96.3% per LA (supplement). The LAs where foreign-born nationals are frequently located are often correlated with areas that report low pentavalent coverage. When combining these data together to estimate the probability of poliovirus circulation, 21 LAs comprise of over 50% of the estimated risk and several of these LAs are located within cities including Manchester, Birmingham and Greater London (Fig. 2 and Table 3). Consequently, if ENV sampling were targeted within catchment areas that cover these LAs, this would be an efficient form of targeted surveillance.

**Table 3.** Summary of the Local Authorities that constitute over 50% of the estimated risk of poliovirus circulation in England and Wales

Local Authority	County	Pentavalent coverage^a^	Associated Water Company^b^	Pr(circulation)	% Total estimated risk	Population size
Birmingham District (A)	West Midlands	94.8^a^	Severn Trent – Minworth	0.905	7.0%	1 073 045
Manchester District (A)	Greater Manchester	95.9^a^	United Utilities – Davyhulme	0.924	5.3%	503 127
Bradford District (A)	West Yorkshire	97.2	Yorkshire Water – Esholt	0.947	4.6%	522 452
Newham (B)	London Borough	94.2^a^	Thames Water – Beckton	0.896	3.8%	307 984
Redbridge (B)	London Borough	95.2^a^	Thames Water – Beckton	0.912	2.6%	278 970
Ealing (C)	London Borough	95.9^a^	Thames Water – Mogden	0.923	2.4%	338 449
Leeds District (C)	West Yorkshire	97.1	Yorkshire Water -	0.945	2.3%	751 485
Waltham Forest (B)	London Borough	92.1^a^	Thames Water – Beckton	0.864	2.2%	258 249
Luton (C)	Luton	96.3	Thames Water	0.932	1.9%	203 201
City of Nottingham (C)	Nottingham	94.7^a^	Severn Trent – Stoke Bardolph	0.904	1.9%	305 680
Hounslow (C)	London Borough	89.7^a^	Thames Water – Mogden	0.830	1.9%	253 957
Brent (C)	London Borough	93.1^a^	Thames Water – Mogden	0.879	1.8%	311 215
Sheffield District (C)	South Yorkshire	96.2	Yorkshire Water	0.929	1.5%	552 698
Slough (C)	Outer London	94.5^a^	Thames Water	0.901	1.5%	140 205
Hillingdon (C)	London Borough	95.1^a^	Thames Water – Mogden	0.911	1.5%	273 936
City of Westminster (B)	London Borough	75.9^a^	Thames Water – Beckton	0.675	1.4%	219 396
Caerdydd – Cardiff (C)	Wales	95.0^a^	Glas Cymru	0.909	1.4%	346 090
Kirklees District (C)	West Yorkshire	98.1	Yorkshire Water	0.963	1.3%	422 458
Barnet (C)	London Borough	92.1^a^	Thames Water – Mogden	0.864	1.3%	356 386
Greenwich (C)	London Borough	93.4^a^	Thames Water – Crossness	0.883	1.2%	254 557
Barking and Dagenham (B)	London Borough	90.5^a^	Thames Water – Beckton	0.840	1.2%	185 911

The parentheses A, B and C refer to the ENV sampling strategies described in the results.

a LAs where the pentavalent coverage is below the national average (96.3%).

b Where possible the likely sewage treatment works is given.

### Estimating the probability of being poliovirus free from surveillance data

The probability of detecting poliovirus through only clinical surveillance if it is circulating is low as clinical disease is a minority of infections. The probability of detecting one infection from clinical surveillance (the combined use of AFP and ENT surveillance) is estimated to be 0.00324 (95% CI 0.00177–0.00481) for wild-type poliovirus and 0.000336 (95% CI 0.000160–0.000641) for VDPVs. The freedom from infection model uses these values along with estimates of poliovirus circulation within each LAs to estimate the sensitivity of each mode of surveillance per month. Using the information available on sampling sensitivity and surveillance activities within England and Wales, the sensitivity of detecting wild-type poliovirus using AFP and ENT surveillance at the specified design prevalence within a given month was estimated to be 0.096 (95% CI 0.055–0.134), and lower for VDPV (0.0111 with 95% CI 0.0053–0.0210).

We explore several scenarios for the use of ENV in England and Wales. ENV surveillance has a differing profile to clinical surveillance as it is sensitive where it is implemented but is limited by the size of the sewage catchment area included in sampling. Implementing monthly ENV in Birmingham, Manchester and Bradford (the LAs with highest risk of importation and circulation, strategy A (Table 3)) the sensitivity of ENV (*CSe*_ENV_) is estimated to be 0.0868 (95% CI 0.0867–0.0869). Sampling in the three high-risk LAs and Beckton (strategy B, where Beckton serves many LAs in London) has an estimated sensitivity of 0.192 (95% CI 0.191–0.193). Performing fortnightly instead of monthly sampling in the same sites would result in only a moderate increase in sensitivity despite a doubling of samples. ENV surveillance capturing LAs that comprise 50% of the total risk (strategy C) would correspond to an estimated sensitivity of 0.32 (95% CI 0.31–0.33), and would consist of 10 ENV samples per month. Including monthly ENV surveillance within Birmingham, Manchester and Bradford would increase the total sensitivity of detecting wild-type poliovirus to 0.174 (95% CI 0.139–0.209) and with the addition of Beckton the sensitivity would increase to 0.270 (95% CI 0.239–0.301), with slightly lower values for VDPV surveillance.

**Fig. 3.**
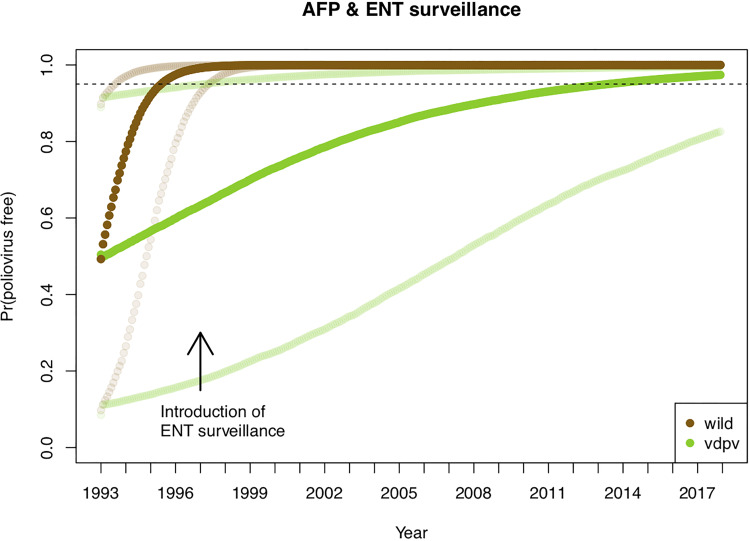
Estimates of the probability of being poliovirus free within England and Wales. The dark brown line is the median estimate and the lighter brown lines are the 2.5 and 97.5 percentile estimates. The arrow indicates when enterovirus surveillance was introduced. The dashed line indicates a 0.95 probability, which was reached by early 1996 for the wild virus analysis (VDPV is shown as a comparator).

We then estimate the probability that England and Wales was free from wild-type poliovirus, given the operating surveillance and the absence of cases or infections, from 1993 to present day. The probability of being poliovirus free increases over time from the date of the last reported case of poliomyelitis in England and Wales in 1993. The introduction of ENT surveillance in 1997 was estimated to moderately improve the rate of increase in the probability of being free from poliovirus due to the higher sensitivity (Fig. 3). There was no noticeable difference in the temporal change in probabilities when different importation risks were assumed or when using the population movements from IPS data *vs.* assuming a constant importation rate. Inclusion of ENV into the estimates for the latter years would not change the estimates of England and Wales being polio free, largely because the estimates are already above 99.9%. A comparison of VDPV is also shown, but it is noted that VDPVs have never been reported in England and Wales. The lower sensitivity of surveillance means that the probability of being infection free increases but at a lower rate. Inclusion of ENV in surveillance improves sensitivity to detect VDPVs, meaning that surveillance will also improve detection of introduced VDPVs.

## Discussion

In the final stages of polio eradication, surveillance for circulation of polioviruses remains essential. The practicalities of surveillance are becoming increasingly challenging owing to the reduced incidence of disease, an increase in the variety of risks that need to be considered and an increasingly connected world that potentially increases risk through population movement. The findings presented here illustrate the potential weaknesses of using clinical surveillance alone to detect poliovirus in England and Wales, and the added benefits of incorporating ENV. Using ENV in an informed, targeted manner has the potential to greatly enhance surveillance for polioviruses, thus expedite detection of importation events.

The approach described here assumes that spatial variation in risk within England and Wales can be quantified using data and used to inform where ENV should be targeted to maximise detection. Use of spatial risk mapping helps prioritise ENV sampling according to risk and estimation of surveillance sensitivity enables comparison of sampling strategies. This has been especially useful in developing the poliovirus ENV strategy within England and Wales. Sampling sewage from the highest risk LAs targets surveillance within areas most likely to be exposed to poliovirus, and sampling within a large London sewage treatment works is advantageous as it covers a considerable proportion of the population with just one ENV sample. Within a pilot scheme implemented between 2016 and 2017, Sabin poliovirus was detected in several samples, illustrating that poliovirus can be detected within a large sewage plant [17]. Sampling in more sites largely out-performs more frequent sampling in the same sites, but may be sensitive to our assumptions on the duration of poliovirus shedding.

Much of the spatial variation in risk is due to movements between England and Wales and countries that have or are currently reporting wild-type and VDPV poliomyelitis cases. We assume that migration at a LA level is similar to the location of foreign-born nationals within the census. Data from IPS supports this assumption, as most residents report visiting friends and family when visiting Afghanistan, Pakistan and Nigeria. There are less data to quantify movements from Laos, DR Congo, Guinea, Myanmar and Syria, which have all reported poliomyelitis cases in recent years. With the emergence of VDPVs in Africa, the risk of importation is likely to have only increased marginally due to the low number of movements between here and England and Wales. Should the incidence of VDPVs increase in Asia (and especially Pakistan which has both ongoing wild-type and VDPV transmission, and much more travel to England and Wales) the risk of importation into the England and Wales will further increase. As VDPVs have a lower symptomatic rate, the addition of ENV to clinical surveillance becomes even more important. Vaccination coverage within LAs influences the likelihood of virus circulation, and ensuring that coverage remains above 90% across communities remains essential. It is therefore a concern that some LAs, especially in London boroughs, consistently report coverage below this value and these are often the same LAs with a higher proportion of foreign-born residents. Risk factors associated with low pentavalent coverage have not been specifically explored in England and Wales, but studies for other vaccines suggest that ethnicity and socio-economic factors are associated with lower coverage [18]. Strategies to improve vaccination rates within these underserved communities should therefore be prioritised.

Estimates of the probability of being infection free are moderately sensitive to assumptions on the probability of importation, which remain uncertain within England and Wales. Visitors to countries that are at risk of poliovirus are recommended to receive a booster of IPV/pentavalent vaccine, and visitors from at-risk countries are required to provide evidence of recent vaccination history as part of the continued Public Health Emergency of International Concern for poliomyelitis. Visitors to Saudi Arabia, as part of religious pilgrimages (Hajj or Umrah) are recommended to receive vaccinations [19]. Consequently, substantial efforts are put in place to reduce the risk of poliovirus importations to England and Wales, but the risk remains, as illustrated by recent importation events within other high-income countries [20, 21].

There are several caveats to the analysis that may warrant further research. We have not considered the risks associated with laboratory release, which are currently considered low, but the relative risks associated with Polio Essential Facilities located in England and Wales will increase as polio eradication approaches the post-certification phase [22]. We do not consider the risks associated with transmission of polioviruses from immune-compromised individuals shedding iVDPVs; despite intensive study there has only been a small handful of transmission events recorded and there is only one reported individual within the UK known to shed iVDPV [23]. Further exploration of ENT surveillance for detecting polioviruses is warranted, as current stool sampling is limited even though the sensitivity of detection is high and the sampling is non-invasive. Populations of unvaccinated adults may pose a risk within specific geographical communities but currently there is little information to rely on. Further details of catchment areas will be needed to select suitable sampling sites and this requires collaboration with water companies. Additionally, the precise sensitivity of an ENV sample is dependent on many factors not considered in the model but described elsewhere [24]. Instead, we included a large range of uncertainty and took this decision because of the lack of data to inform calculations but this can be revisited should the data become available. The specified design prevalence affects the estimates of sensitivity and as eradication approaches a more stringent design prevalence may be warranted. Methodological developments may be required to validate the approach, such as simulation. With these caveats in mind, it should be noted that the exact risk probabilities may be uncertain but the relative difference between LAs and mode of surveillance should still hold.

## Conclusion

Surveillance for poliovirus is becoming increasingly complex owning to the different modes of surveillance, and the changing risk of poliomyelitis. This research attempts to quantify the variation in poliovirus risk in a disease-free setting, and use of these estimates to compare different modes of surveillance. ENV surveillance will improve the sensitivity of surveillance, thus supporting the certification phase of polio eradication.

## Supplementary material

For supplementary material accompanying this paper visit http://dx.doi.org/10.1017/S0950268820001004. click here to view supplementary material
