# Supplementary material for: Surveillance optimisation to detect poliovirus in the pre-eradication era: a modelling study of England and Wales
Source: Epidemiol Infect. 2020 May 13;148:e157. doi: 10.1017/S0950268820001004 (PMC7379320; doi:10.1017/S0950268820001004)
Supplement: Supplementary file 1 [file S0950268820001004sup001.docx]

# Supplementary Material for “Surveillance Optimization to Detect Poliovirus in the Pre-Eradication Era: a Modelling Study of England and Wales”

1. **Vaccination coverage of the pentavalent vaccine**

**Figure 1.** Average pentavalent coverage in children under 5 years of age by Local Authority within A) England and Wales and B) London Boroughs, 2011-2016. Local authorities that report coverage below 90% are highlighted.

1. **Water companies and likely sewage treatment works serving high risk local authorities**

***Severn Trent water company***

Severn Trent water company serves cities such as Birmingham, Nottingham, Leicester and Wolverhampton, as described on their website (https://www.severntrent.com/content/dam/stw-plc/water-resource-zones/WRMP-main-narrative.a.pdf). Minworth sewage treatment works (in Sutton Coldfield) serves the area of Birmingham City. The sewage treatment works of Stoke Bardolph is the likely treatment works that serves the Nottingham area.

***United Utilities***

The Greater Manchester area is served by United Utilities (<https://www.unitedutilities.com/help-and-support/wastewater-services/>). The treatment works that serves most of the Manchester are is the Davyhulme treatment works.

***Yorkshire Water***

Yorkshire water serves Bradford, with Esholt treatment works being the main sewage treatment for this area (<https://www.yorkshirewater.com/waste-water-treatment-services/>). For Leeds, the likely sewage treatment facility is Knostrop Wastewater Treatment Works. For Sheffield the likely sewage treatment works is Woodhouse Mill Sewage Treatment Works.

***Thames Water***

Thames water is the company that serves the Greater London area and some additional counties surrounding London. There are three main sewage treatment works; Beckton, Crossness and Mogden. The catchment of these treatment works has been well described by Thames Water (see figure), and can be used to approximate which would be sampled if carrying out surveillance of each population within the Local Authority.

***Glas Cymru***

Glas Cymru is the water treatment company for Wales. The likely sewage treatment works for Cardiff is Llwyn Onn Sewage Works.

**
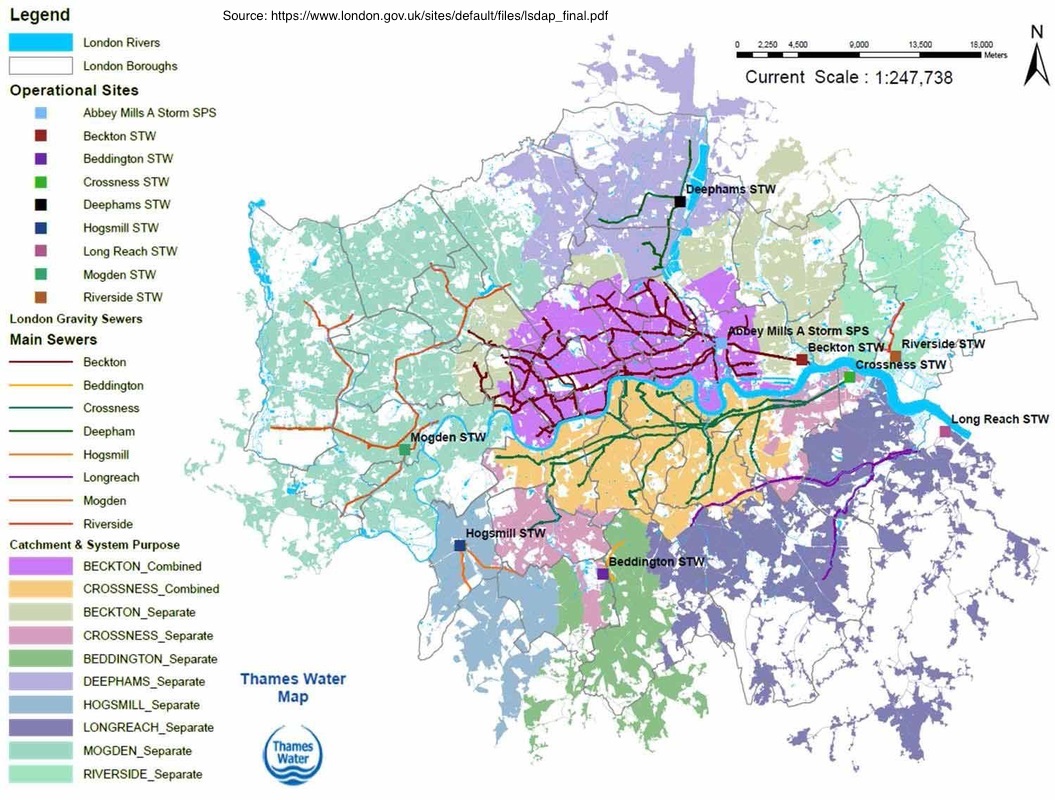
**

Figure 2. Converging sewer network of Great London overlaid onto the London Boroughs (from Thames Water).

1. **Estimation of the spatial variation in the potential for poliovirus circulation in England and Wales**

The probability of local poliovirus circulation (herein referred to as poliovirus circulation) is estimated. Within this context we are interested in circulation that may result in a poliomyelitis case. The basic reproduction number (R_0 -_ the average number of secondary cases from one infected person within a totally susceptible population) is useful measure of transmissibility of an infectious disease. When R_0_ is greater than 1 the introduction of an infected individual may result in a large outbreak and if R_0_<1 a large outbreak is unlikely. For vaccines with sterilizing immunity the probability of a major epidemic is given by s=$1-1/{{(R}_{0}(1-c))}$ where $c$ refers to the proportion of the population immunised. Rather than sterilising immunity, the IPV (which is part of the pentavalent vaccine) reduces the infectiousness and duration of infectiousness of immunised individuals,^1^ so the probability of a major epidemic requires some adaptation; $s=1-1/{(R_{0}((1-c)+chd))}$ where $h$ is the relative infectiousness of immunised individuals and $d$ is the relative change in the duration of infectiousness.

The probability of major epidemic was estimated for each LA in England and Wales. To estimate the potential for poliovirus circulation the probability of circulation and the number of importation events ($M_{j}$) are combined assuming a binomial process. The risk for LA $i \{i=1,\ldots,N\}$ was estimated by summing over all locations considered to be a potential source (*j*);

$R_{i}=1-\sum_{j=1}^{N} {(1-s_{i})}^{M_{j}}$. equation 2.1

For ENV surveillance in England and Wales to be optimised, sampling should be prioritised within sewage catchment areas with LAs with the highest risk. Wastewater treatment in England and Wales consists of eleven companies that manage regions similar to counties. Analysis of the spatial variation in poliovirus risk does not fully align with the location of sewage catchment areas but we provide an indication of the LAs and corresponding water company, and where possible the sewage treatment works that should be sampled to capture wastewater from each LA. The risk of poliovirus circulation is assumed to vary between wild and VDPVs based on just the location of reported cases and migration between England and Wales and these countries.

The estimated number of importations of either wild or VDPV poliovirus (*M*) is a function of the number of number of visitors to (N(i,j)) and from each country (N(j,i)), their vaccination status (v(i) and v(j)), and the incidence of WPV and VDPVs within each country.

$M_{WPV}\left( j \right)=N\left( i,j \right).I_{WPV}\left( j \right).(1-\nu\left( i \right))+N\left( j,i \right).I_{WPV}\left( j \right).(1-\nu\left( j \right))$ equation 2.2

$M_{VDPV}\left( j \right)=N\left( i,j \right).I_{VDPV}\left( j \right).(1-\nu\left( i \right))+N\left( j,i \right).I_{VDPV}\left( j \right).(1-\nu\left( j \right))$ equation 2.3

The total number of movements are $M_{j}=M_{WPV,j}+M_{VDPV,j}$.

1. **Estimation of surveillance sensitivity**

The methods for estimating the sensitivity of each mode of surveillance are first described.

For one poliovirus infection the sensitivity of detection through AFP surveillance (SeU_AFP_) is estimated by taking the product of each step in the detection pathway (ie. from the probability of an infection being symptomatic through to the probability of the diagnostic test being positive, see Figure 1). If the risk of infection is constant across all locations, the estimate of sensitivity remains relatively simple. However, if the risk of infection varies across settings, it is intuitive that sampling within high risk settings would be preferable to sampling in low risk settings, and so the sensitivity needs to be adjusted. Acknowledging that this adjustment is an approximation,^2^ we can use estimates of relative risks. The effective probability of infection (EPI) is used to account for variation in poliovirus risk across the LAs. The EPI combines the adjusted risk and the herd prevalence, ${EPI}_{i}={AR}_{i}.P_{h}^{*}$. The relative risks (RR), estimated in the previous section, and the proportion of the population (PrP) are used to calculate the adjusted risk (AR), by solving the simultaneous equations;

$\frac{\mathrm{AR}_{i}}{\mathrm{AR}_{j}}=\frac{\mathrm{RR}_{i}}{\mathrm{RR}_{j}}$ equation 3.1

$\mathrm{AR}_{1}.\mathrm{PrP}_{1}+\ldots+\mathrm{AR}_{n}.\mathrm{PrP}_{n}=1$. equation 3.2

High risk LAs will have an adjusted risk above 1.00 and consequently increased surveillance within these settings would have a more rapid improvement in the sensitivity of the entire system. Combining these elements together, the sensitivity of detection through AFP surveillance is;

$$\mathrm{SeU}_{AFP,i}={EPI}_{i}.\Pr\left( \mathrm{case}_{\mathrm{AFP}} \right).\Pr\left( \mathrm{notification}_{\mathrm{AFP}} \right).\Pr\left( \mathrm{sample}_{\mathrm{AFP}} \right).Pr(\mathrm{test}_{\mathrm{AFP}})$$

To calculate the sensitivity of AFP within each LA in a given month (SSe_AFP,i_), we assume that all individuals (n) within each LA are included in surveillance;

$$\mathrm{CSe}_{AFP,i}=1-{(1-\mathrm{SeU}_{AFP,i})}^{n_{i}}$$

Similar calculations are done for enterovirus (CSe_ENT,i_) and environmental surveillance (CSe_ENV,i_). All probabilities include uncertainty which is carried through the calculations and the mean and 95% credible intervals are given. See section 5 for a description of how estimates for each element of surveillance were derived.

We then want to estimate the sensitivity across the entire system for each mode of surveillance. First we need to combine estimates LAs for each surveillance system;

$$\mathrm{CSe}_{\mathrm{AFP}}=1-\prod_{i=1}^{M} {CSe}_{AFP,i}$$

Then these values are used to estimate the surveillance sensitivity of the entire system; $CSe=1-(\left( 1-{CSe}_{AFP} \right)\left( 1-{CSe}_{ENT} \right)\left( 1-{CSe}_{ENV} \right))$.

As with other high-income countries sewage collects into a catchment area so maps from water companies can be used to determine the extent of population coverage if ENV is initiated at a specific sewage works. Composite samples are taken from the inlet of the sewage works and previous research suggests they have a high sensitivity to poliovirus if an individual is shedding within the last week.^3^ Shedding studies have illustrated a high sensitivity of detecting poliovirus from just one composite sample and so the sensitivity of ENV is largely influenced by the frequency of sampling. If an individual sheds poliovirus for approximately 16 days,^3^ poliovirus could be detected within sewage for up to 23 days from one individual, resulting in a monthly sampling frequency corresponding to a detection probability of (23+1)/30=0.80. The duration of shedding in IPV vaccinated individuals is lower,^1^ but if more than one individual is shedding poliovirus the probability of detection would increase. To account for the known variation in shedding and uncertainty in the number of shedders the probability of detection is given wide confidence intervals and monte-carlo simulation is used to sample from these distributions (Table 3). Fortnightly sampling within one location would increase the detection probability to 1.00. Sensitivity of detection is thought to reduce with increasing sewage flow^4^ and so sensitivity may be lower in sewage sites processing wastewater from a large (>100,000) population, but empirical studies that test this are lacking.

1. **Estimation of the probability of being polio-free**

Using the principal of the negative predictive value of a test, and assuming that testing has 100% specificity, the probability of being infection free within a given month (*t*) is $P({free}_{t})={(1-P)}/{(1-P.{CSe}_{t})}$, where *P* is the prior for being infected and $CSe$ refers to diagnostic sensitivity, which in this case is the sensitivity of the surveillance system. Each prior probability is the $P({free}_{t})$ for the previous time period, which is a combination of being free during the previous time period and accounting for the probability of introduction. We also need to account for poliovirus introduction for each calendar month ($P({Intro}_{t})$). The probability of being infection free within a calendar month is then given as;

$P\left( {free}_{t-1} \right)=1-\left( P\left( {infect}_{t-1} \right)+P\left( {intro}_{t-1} \right)-P\left( {infect}_{t-1} \right).P\left( {intro}_{t-1} \right) \right)$ equation 4.1

$P\left( {free}_{t} \right)=(1-P({free}_{t-1}))/(1-P\left( {free}_{t-1} \right).{CSe}_{t}$ equation 4.2

Different sampling strategies are considered for ENV surveillance. The rate of poliovirus introduction is unknown, consequently the rate is estimated from approximating population movements to and from countries reporting poliomyelitis cases and the probability of shedding. From the International Passenger Survey (Figure 3) we can approximate the numbers of individuals that travel to and from countries reporting polio cases and combine this with the reported incidence of wild and VDPV within each country to provide an upper estimate of the rate of introduction. Low (1x10^-4^), medium (1x10^-3^) and high (5x10^-3^) probabilities of poliovirus introduction per month were used to account the true value being unknown.


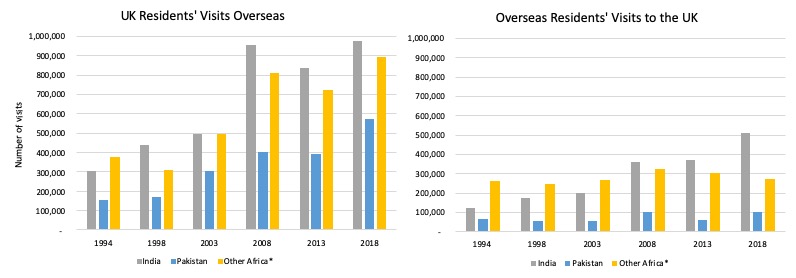


Figure 3. Estimates of the number of visits to and from countries that have reported poliomyelitis cases (IPS data).

1. **Justification for probabilities used in the scenario tree modelling**

In most probabilities estimated below, a mean and 95% confidence intervals are provided from the available data. These values were inputted to the model by specifying a beta distribution with parameters $\alpha$ and $\beta$. Within the software R, the specific parameter values that correspond to the specified mean and 95% confidence intervals were provided using the library ‘prevalence’.

Acute Flaccid Paralysis (AFP) Surveillance

- Pr(case_AFP_): Of individuals infected with poliovirus Nathanson et al.^5^ report that the probability of a clinical case varies by serotype; 0.005, 0.0005 and 0.0009 for serotypes 1, 2, and 3 respectively. A beta distribution with mean 0.005 and 95% CI 0.0005-0.009 encapsulates these estimated probabilities and the possible uncertainty in the values. As we estimate the sensitivity for wild poliovirus and vaccine-derived poliovirus separately, we use the estimates for serotype 1 and 2.
- Pr(notification_AFP_): A case of AFP caused by poliovirus is an acute condition where it is very likely in a high income setting that healthcare will be sought. However, there is little data to inform this probability so we have taken precautionary principle of using knowledge from all cases of AFP (including Guillian-Barre syndrome) within England and Wales.
- Pr(sample_AFP_): Stool sampling is recommended as part of the clinical investigation of all AFP cases. Within Salisbury et al. ^6^ of 0.54 of AFP cases had stool samples. Since this time clinical sampling has mostly likely improved, so we select a mean of 0.8 with confidence intervals ranging from 0.5-0.95.
- Pr(test_AFP_): Samples to be tested for poliovirus undergo rigorous laboratory testing where PCR is used to determine the presence of enterovirus in stool, CSF or throat swabs. Following a positive PCR virus culture, intratypic differentiation and sequence analysis will be carried out as per the WHO protocol. The testing is highly sensitive with reported sensitivities above 0.95 for ~100 RNA copies per microlitre for ITD 5.0 which is a similar protocol to that used in testing within England and Wales^7^. We specify a high probability of detection with a mean of 0.97 and 95% CI of 0.95-1.00.

Enterovirus (ENT) Surveillance

- Pr(case_ENT_): Conditions such as aseptic meningitis are also clinical indicators of poliovirus infection in addition to acute flaccid myelitis. There are fewer reports of the probability of developing aseptic meningitis, but Mehndiratta et al estimate that meningitis occurs in about 1% of cases,^8^ although no distinction is made between serotype. Additionally, during a serotype 3 polio outbreak in Finland, nine cases of paralytic poliomyelitis were reported and one case of aseptic meningitis, which could be used to suppose a 9:1 ratio of paralytic polio to aseptic meningitis.^9^ Confidence intervals 0.01-0.05 were used to account for uncertainty in this estimate.
- Pr(notification_ENT_): Clinical disease associated with enterovirus infection is used as a proxy for the notification rate that might be associated with aseptic meningitis caused by poliovirus infection. Estimates are not available but it is assumed that health-seeking behaviours would be similar to that for poliovirus infection
- Pr(sample_ENT_): For clinical disease associated with enterovirus infection, Majumdar et al. ^9^ provide a description of current surveillance activities and report that a large proportion of clinical cases have microbiological samples (stool and CSF) that would enable culture of poliovirus. We assume an average probability of collecting a sample of 0.8 with wide confidence intervals (95% CI 0.5-0.95 to account for the large uncertainty in the estimate.
- Pr(test_ENT_): The microbiological protocol for clinical samples via ENT surveillance is the same as AFP diagnostics, so the same probabilities are used.

Environmental (ENV) Surveillance

- Pr(shedding_ENV_): We assume that all individuals infected with poliovirus shed poliovirus in stool.
- Pr(catchment_ENV_): As sewerage systems in England and Wales consist of a convergent sewer system, we can assume that all waste will eventually reach a sewage processing plant. Each local authority is assumed to have at most one sewage treatment plant that can be sampled for ENV surveillance. In many areas, for example London, one sewage treatment plant serves several local authorities. Within the framework, if a local authority is assumed to be sampled during ENV surveillance, we assume a probability of 0.8 that the infected individuals would be sampled (to account for time spent outside of the sewage catchment area).
- Pr(sample_ENV_): Sampling of wastewater is carried out using a composite sampler, which takes small samples of the wastewater over a 24 hour period ^9^. If an individual sheds poliovirus for approximately 16 days,^3^ poliovirus could be detected within sewage for up to 23 days from one individual, resulting in a monthly sampling frequency corresponding to a detection probability of (23+1)/30=0.80.
- Pr(test_ENV_); The microbiological protocol for clinical samples via ENV surveillance is similar as AFP diagnostics, with an added concentration step (2-phase separation) ^9^. It is not thought that the concentration step reduced detection sensitivity, but the large amount of contamination from mixed sewage may reduce culture success of poliovirus.

References

1. Hird, T. R. & Grassly, N. C. Systematic review of mucosal immunity induced by oral and inactivated poliovirus vaccines against virus shedding following oral poliovirus challenge. *PLoS Pathog.* **8**, (2012).

2. Cameron, A., Njeumi, F., Chibeu, D & Martin, T. RISK-BASED DISEASE SURVEILLANCE.

3. Lodder, W. J. *et al.* Feasibility of quantitative environmental surveillance in poliovirus eradication strategies. *Appl. Environ. Microbiol.* **78**, 3800–3805 (2012).

4. Hovi, T., Stenvik, M., Partanen, H. & Kangas, A. Poliovirus surveillance by examining sewage specimens. Quantitative recovery of virus after introduction into sewerage at remote upstream location. *Epidemiol. Infect.* **127**, 101–106 (2001).

5. Nathanson, N. & Kew, O. M. From emergence to eradication: the epidemiology of poliomyelitis deconstructed. *Am. J. Epidemiol.* **172**, 1213–1229 (2010).

6. Salisbury, D. M., Ramsay, M. E., White, J. M. & Brown, D. W. Polio eradication: surveillance implications for the United Kingdom. *J. Infect. Dis.* **175 Suppl 1**, S156-159 (1997).

7. Gerloff, N. *et al.* Diagnostic Assay Development for Poliovirus Eradication. *J. Clin. Microbiol.* **56**, (2018).

8. Mehndiratta, M. M., Mehndiratta, P. & Pande, R. Poliomyelitis. *The Neurohospitalist* **4**, 223–229 (2014).

9. Simonen, ML, Roivainen, M, Iber, J, Burns, C & Hovi, T. Outbreak of poliomyelitis in Finland in 1984-85-Re-analysis of viral sequences using the current standard approach. *Virus research.* 2010. 147(1); 91-97. doi: 10.1016/j.virusres.2009.10.012

10. Majumdar, M. *et al.* Isolation of Vaccine-Like Poliovirus Strains in Sewage Samples From the United Kingdom. *J. Infect. Dis.* **217**, 1222–1230 (2018).
